# whISOBAX^TM^ Inhibits Bacterial Pathogenesis and Enhances the Effect of Antibiotics

**DOI:** 10.3390/antibiotics9050264

**Published:** 2020-05-19

**Authors:** Reuven Rasooly, Hwang-Yong Choi, Paula Do, Gianluca Morroni, Lucia Brescini, Oscar Cirioni, Andrea Giacometti, Emmanouil Apostolidis

**Affiliations:** 1 U.S. Department of Agriculture, Agricultural Research Service, Albany, CA 94710, USA; paula.do@usda.gov; 2 Department of Chemistry and Food Science, Framingham State University, Framingham, MA 01701, USA; kelolo123123@gmail.com; 3 Department of Biomedical Sciences and Public Health, Marche Polytechnic University, 60121 Ancona, Italy; lucia.brescini@ospedaliriuniti.marche.it (L.B.); o.cirioni@univpm.it (O.C.); Andrea.Giacometti@ospedaliriuniti.marche.it (A.G.)

**Keywords:** biofilm inhibition, staphylococcal enterotoxin inhibition, antibacterial, witch hazel, whISOBAX, checkerboard assay, isobologram analysis

## Abstract

As bacteria are becoming more resistant to commonly used antibiotics, alternative therapies are being sought. whISOBAX (WH) is a witch hazel extract that is highly stable (tested up to 2 months in 37 °C) and contains a high phenolic content, where 75% of it is hamamelitannin and traces of gallic acid. Phenolic compounds like gallic acid are known to inhibit bacterial growth, while hamamelitannin is known to inhibit staphylococcal pathogenesis (biofilm formation and toxin production). WH was tested in vitro for its antibacterial activity against clinically relevant Gram-positive and Gram-negative bacteria, and its synergy with antibiotics determined using checkerboard assays followed by isobologram analysis. WH was also tested for its ability to suppress staphylococcal pathogenesis, which is the cause of a myriad of resistant infections. Here we show that WH inhibits the growth of all bacteria tested, with variable efficacy levels. The most WH-sensitive bacteria tested were *Staphylococcus epidermidis,* *Staphylococcus aureus*, *Enterococcus faecium* and *Enterococcus faecalis*, followed by *Acinetobacter baumannii*, *Klebsiella pneumoniae*, *Escherichia coli,* *Pseudomonas aeruginosa*, *Streptococcus agalactiae* and *Streptococcus pneumoniae*. Furthermore, WH was shown on *S. aureus* to be synergistic to linezolid and chloramphenicol and cumulative to vancomycin and amikacin. The effect of WH was tested on staphylococcal pathogenesis and shown here to inhibit biofilm formation (tested on *S. epidermidis*) and toxin production (tested on *S. aureus* Enterotoxin A (SEA)). Toxin inhibition was also evident in the presence of subinhibitory concentrations of ciprofloxacin that induces pathogenesis. Put together, our study indicates that WH is very effective in inhibiting the growth of multiple types of bacteria, is synergistic to antibiotics, and is also effective against staphylococcal pathogenesis, often the cause of persistent infections. Our study thus suggests the benefits of using WH to combat various types of bacterial infections, especially those that involve resistant persistent bacterial pathogens.

## 1. Introduction

Community and healthcare-acquired bacterial infections (CAI and HAI) are becoming harder to treat because of bacterial resistance to antibiotics [1]. In the United States for example, each year about 2 million HAIs occur, which result in about 100,000 deaths. The cost to treat such infections is about $40 billion [2,3,4]. According to the Center of Disease Control (CDC) in 2014, of the infected individuals, pneumonia (21.8%) and surgical site infections (21.8%) were the leading infections, followed by gastrointestinal infections (17.1%), urinary tract infection (12.9%) and primary bloodstream infection (9.9%) [5]. Bacteria most frequently associated with HAIs include Gram-positive bacteria, such as *Staphylococcus aureus, Staphylococcus epidermidis* and *Enterococcus faecalis*, as well as Gram-negative bacteria, including *Escherichia coli, Klebsiella pneumoniae, Proteus mirabilis* and *Pseudomonas aeruginosa*. *Clostridium difficile* infections are also common in healthcare settings, but those mostly result from extensive antibiotic use needed to treat initial infections caused by other bacteria [6].

Some bacteria are behaviorally resistant to antibiotics through formation of biofilms, which are like fortresses protecting bacteria and other microorganisms from environmental stressors. Biofilms are communities of microorganisms that can attach, e.g., to host cells or to medical devices and have been implicated in nonhealing chronic, persistent infections. Biofilms are surrounded by extracellular polymeric substances (EPS) [7], mainly consisting of polysaccharides, extracellular DNA and proteins made by biofilm cells, which help to protect them from external threats, like the host’s immune response and antimicrobials [8].

Biofilms enhance resistance and persistence profiles of the organisms involved. In a biofilm, bacteria are more likely to act collectively to benefit themselves, often at the expense of the host [8]. Biofilms have been associated with a number of chronic infections. For example, *S. aureus* is part of a normal healthy microbiome of the skin and mucus membranes. But once their numbers increase and they reach a certain quorum (as is the case in a biofilm), these bacteria secrete many types of toxins. These toxins include, e.g., proteases that disrupt host tissue, or enterotoxins and toxic shock syndrome toxin (TSST) that interfere with the host’s immune response and can cause sepsis and death [9].

Chronic wound infections are often associated with biofilms containing staph species, and annually in the US, these infections result in over 100,000 amputations [10]. Chronic lung infections are often associated with biofilms formed by *P. aeruginosa* in the lungs of cystic fibrosis (CF) patients, and colonization is often persistent through the lifetime of the patient [11], leading to chronic inflammation and lung tissue damage [12]. Chronic infections persist despite the aggressive use of antibiotics. Antibiotic use can lead to disruption of the normal microflora, potentially giving rise to other health issues, like the rise in secondary *Clostridium difficile* infections causing antibiotic-associated diarrhea. According to the CDC, *C. difficile* has become the most common microbial cause of HAIs in U.S. hospitals, resulting in thousands of deaths and $4.8 billion each year in excess health care costs for acute care facilities alone [6].

It is thus important to enhance bacterial sensitivity to antibiotics, thereby reducing the need for extensive use of antibiotics, while combating resistant persistent infections.

Plants synthesize a diverse array of secondary metabolites (phytochemicals) used by the plant for defense mechanisms, and have antimicrobial properties [13,14,15,16]. Witch Hazel (*Hamamelis virginiana*) contains multiple phenolic compounds, such as gallic acid, gallocatechin and epigallocatechin. These compounds bind to the bacterial cell membrane and cause bacterial cell disruption [16,17]. Importantly, witch hazel contains hamamelitannin (2′,5-di-*O*-galloyl-*d*-hamamelose), which is a low-molecular-weight phenolic compound. Hamamelitannin (HAMA) has been shown to act as a quorum-sensing inhibitor in staphylococci, inhibiting bacterial virulence, i.e., inhibiting biofilms from being formed and toxins from being produced [18,19,20,21,22,23]. An extract rich in phenolic compounds (whISOBAX, StaphOff Biotech Inc., Hopkinton, MA, USA) was tested for its antibacterial activity against Gram-positive and Gram-negative bacteria. Its synergism with antibiotics as well as its inhibitory effect on *S. epidermidis* biofilm and *S. aureus* toxin production was also tested.

## 2. Methods

### 2.1. Bacteria

*S. epidermidis* ATCC 35984/RP62A, *S. aureus* USDA strain, *S. aureus* ATCC 29213, *S. aureus* MRSA ATCC 43300, *S. pneumoniae* ATCC49619, *S. agalactiae* 1357, *E. coli* ATCC 25922, *P. aeruginosa* ATCC 27853, *K. pneumoniae* ATCC 700603, *A. baumannii* ATCC 19606, *E. faecium* 64/3, *E. faecalis* ATCC 29212. Bacteria were grown in Tryptic Soy Broth (TSB) or in cation-adjusted Mueller-Hinton broth (MH) as indicated. Streptococci were grown in MH broth supplemented with 3% laked horse blood.

### 2.2. Test Formulations

whISOBAX (WH), a witch hazel extract rich in phenolic compounds, containing 49 mg/mL dry weight, was supplied by StaphOff Biotech Inc. Hopkinton, MA, USA. Ciprofloxacin (110 µg/mL) +/− WH (5%) was supplied by Hopkinton Drug, MA, USA. Unless otherwise noted, all other chemicals were purchased from Sigma-Aldrich Co. (St. Louis, MO, USA).

### 2.3. Stability of Hamamelitannin (HAMA) and Gallic Acid (GA) Content in whISOBAX by Column Chromatography

whISOBAX was analyzed by High-Pressure Liquid Chromatography (HPLC) and HAMA content was determined by comparison to a HAMA and GA standards, according to Wang et al. [24] with some modifications. Column used was Durashell reverse phase C18 (Agilent Technologies, Santa Clara, CA, USA) 3 µm, 100 Ǻ, 4.6 × 50 mm column. Solvents used for separation were 0.1% (*v*/*v*) trifluoroacetic acid (TFA) in water (eluent A) and 0.1% (*v*/*v*) TFA in 100% acetonitrile (eluent B). The gradient used was: 0–2 min, 5% B; 2–10 min, linear gradient to 40% B, which was maintained for 3 additional min. The HPLC system (Agilent 1200) was used with a variable wavelength Detector. The content of HAMA and GA in whISOBAX were confirmed by comparing the retention time and absorbance spectrum with standards.

### 2.4. Minimal Inhibitory Concentrations (MICs)

The minimal inhibitory concentration (MIC) of WH on multiple bacterial species was determined by microdilution assays, where overnight bacterial cultures were first adjusted to 5 × 10^5^ CFU/mL in cation-adjusted MH broth, unless indicated otherwise. Bacteria were then treated with two-fold dilutions of each test material. After overnight incubation at 37 °C, MICs were recorded as the lowest drug concentration that resulted in complete inhibition of visual bacterial growth and by optical density (OD 600 nm). Cells in broth only were used as positive controls. The same solutions incubated without cells were used as background values.

### 2.5. Synergy Testing

The synergistic potential of WH and antibiotics was determined by isobologram analysis of checkerboard microdilution assays. Bacteria (diluted to 5 × 10^5^ CFU/mL) were placed in polypropylene 96-well plates (Falcon, Corning, NY, USA) containing two-fold serial dilutions of WH and antibiotics in MH broth. The plates were incubated overnight at 37 °C and the MIC values were determined by visual inspection, and by optical density analysis at 630 nm. Control plates containing test materials in broth with no bacteria were used as background values. Positive controls included bacteria in broth and respective solvents only. The fractional inhibitory concentrations (FICs) were calculated by dividing the MICs of the drug combination by the individual drug. The sum (Σ FICs) of FIC values of the two test materials were used to indicate if their interaction is synergistic (≤0.5), cumulative (>0.5 to <1.0), indifferent (1.0≤ to 4.0), or antagonistic (>4.0) [25,26,27].

### 2.6. The Effect of WH and Ciprofloxacin on S. epidermidis Growth and Biofilm Formation

*S. epidermidis* strain RP62A was grown to the early exponential phase of growth in TSB. Cells were then plated (~2 × 10^4^ CFU per well) in a 96-well polystyrene plate (Falcon, Corning, NY, USA) and incubated with increasing dilutions of test solutions to a final volume of 200 µL per well for about 18 h at 37 °C. Optical density of 630 nm or OD600 nm was measured using a microtiter plate reader (BioTek, Winooski, VT, USA). Colony-forming units (CFUs) were determined by plating samples on Tryptic Soy Agar (TSA) plates and incubating overnight at 37 °C. The MIC was determined as the lowest concentration resulting in observable colonies. The minimal bactericidal concentration (MBC) was determined as the lowest concentration that resulted in no observable colonies. All experiments were performed in triplicate. Cells in TSB alone or TSB with relevant solvents were used as positive controls. The same solutions incubated without cells were used as background values.

To test for biofilm, the nonadherent cells were removed. Remaining attached bacteria (biofilm) were gently washed 3 times with PBS, fixed with ethanol, and then air-dried. Filtered 0.2% crystal violet in 20% ethanol was added to attached cells and the unbound stain was removed with water. The plates were air-dried and the dye was solubilized with 0.1% SDS in a total volume of 200 µL. Biofilm was determined at OD 630 nm.

The value of initial biofilm was determined by fixing sample wells with ethanol instead of exposure to test solutions.

### 2.7. Staphylococcal Enterotoxin A (SEA) Production

*S. aureus* strain USDA was grown as described above for *S. epidermidis*. SEA production was measured by sandwich ELISA as described [28,29]. Specifically, cells grown with test solutions (see above) were removed by centrifugation and supernatants collected. To detect SEA in the supernatants, the capture antibody was a sheep anti-SEA IgG (Toxin Technology, Sarasota, FL, USA) and the detection antibody was a sheep anti-SEA Horse Radish Peroxidase (HRPO) (Toxin Technology, Sarasota, FL, USA). Capture antibody was incubated overnight at 4 °C in coating buffer (0.01 M NaHCO_3_, 0.1 M Na_2_CO_3_) at a final concentration of 10 µg/mL and 100 µL/well in a microtiter 96-well plates (Greiner, Monroe, NC, USA). Wells were washed 3 times with PBST (PBS containing 0.05% Tween-20), and the same solution (100 µL/well) was used for blocking wells for 15 min at room temperature (RT). One hundred microliters of each sample supernatant were added and incubated for 2 h at 37 °C. Plates were washed 3 times with PBST. Capture antibody, diluted in PBST, were added (100 µL/well), and incubated for 1 hr. at 37 °C. Plates were washed 5 times with PBST. One hundred microliters of 3,3′,5,5-tetramethylbenzidine chromogen solution (Invitrogen, Carlsbad, CA, USA) substrate was added, and 0.3 HCl (50 µL/well) was added to stop the reaction. Absorbance was measured at 450 nm in a microplate reader (BioTek, Winooski, VT, USA) and expressed as 10× OD measured. Increasing amounts of SEA (1 µg/mL to 10 ng/mL) was used as a standard curve.

### 2.8. Statistical Analysis

Experiments were done in triplicates and their averages presented. Standard deviation values were calculated using the Microsoft Excel “n-1” method. Significant differences between two treatment groups was calculated using Microsoft Excel two-tailed Student’s *t*-test and *p* < 0.05 was considered as a significant difference. A one-way analysis of variance (ANOVA) was performed using the Holm–Sidak method for pairwise comparisons with an overall significance level of *p* < 0.05 (Systat Software, Inc., San Jose, CA, USA).

## 3. Results

### 3.1. Stability of Hamamelitannin (HAMA) and Gallic Acid in whISOBAX (WH)

**Figure 1:**
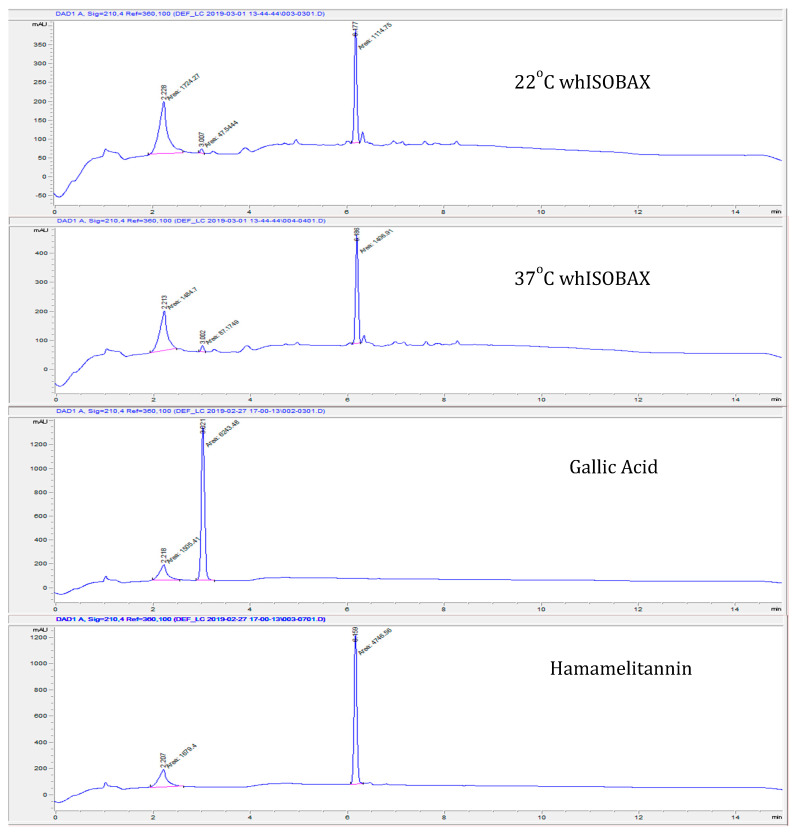
Stability of hamamelitannin (HAMA) and gallic acid in whISOBAX (WH); reverse phase HPLC analysis. WH (StaphOff Biotech Inc, Hopkinton, MA USA) that was incubated at 22 °C or 37 °C for 60 days, HAMA and gallic acid standards (Sigma-Aldrich, MO, USA) were applied to Durashell reverse phase C18 column in water containing 0.1% TFA. Bound material was eluted with an acetonitrile gradient. The HAMA and gallic acid content in WH were determined by comparing the retention time and absorbance spectrum with the standards.

The total phenolic content of WH (50 mg/mL dry weight) was determined to be 12.66 mg/mL Gallic Acid Equivalent (GAE), where 75% of that is due to HAMA [29]. The remaining 25% is due to other phenolic compounds naturally present in witch hazel extract, such as gallic acid [24]. WH was analyzed by reverse phase HPLC, and HAMA and gallic acid content was determined by comparison with their standards. As shown in Figure 1, a single primary peak (eluted at about 25% acetonitrile) is evident at 210 nm, which was determined to be HAMA by comparison of absorbance profile to HAMA standard and confirmed by LCMS analysis [29]. A minor peak (eluted at about 10% acetonitrile) was determined to be gallic acid, by comparison of absorbance profile to gallic acid standard. The stability of whISOBAX was tested by incubating samples at 22 °C or 37 °C and comparing their efficacy and HPLC profiles after 2 months. As shown in Figure 1, HPLC profile (and efficacy, not shown) remained the same after a 2-month incubation period, indicating its stability under these conditions.

### 3.2. The Effect of WH on the In Vitro Growth of Clinically Relevant Bacteria

Bacteria were grown with increasing concentrations of WH, and MICs were determined by broth microdilutions. As shown in Table 1, various degrees of sensitivity to WH were observed by various bacterial species, with mean MICs ranging from ~26–4000 µg/mL (dilution factors of ~1/10 to ~1/2000). The most sensitive were staphylococci and enterococci.

### 3.3. whISOBAX Can Enhance the Antibacterial Effect of Antibiotics

**Figure 2:**
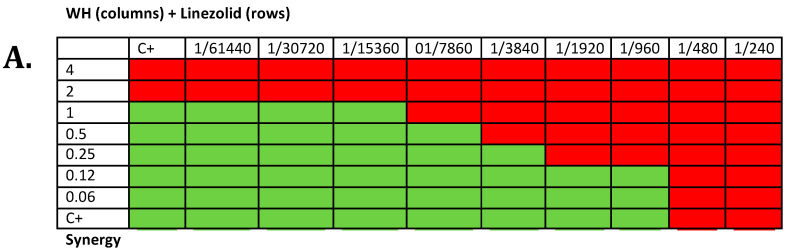

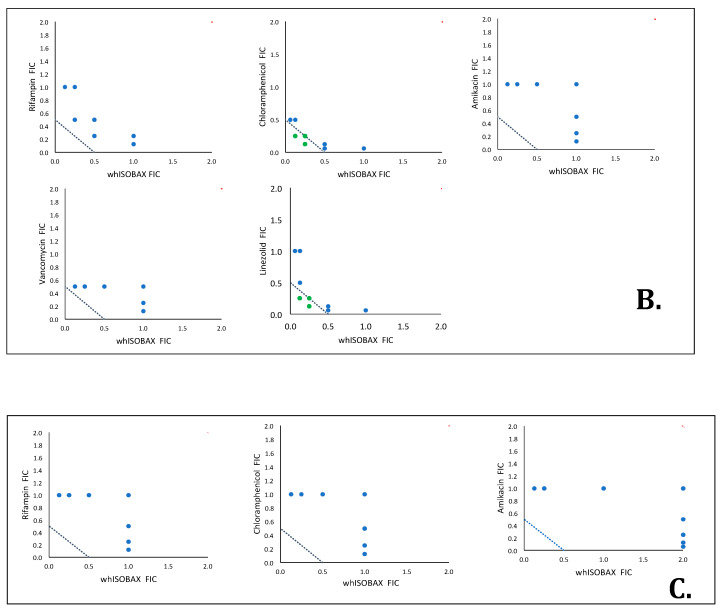
(**A**) Checkerboard assay of WH and linezolid against *S. aureus*; bacteria were grown in 96-well plates with increasing amounts of WH and/or antibiotic overnight at 37 °C, and cell density was determined. Wells in which there was visible bacterial growth are presented as green, while wells with no bacterial growth are presented as red. WH concentrations are expressed as a final dilution of initial extract (1/240 to 1/61,440). Antibiotic concentrations are expressed as mg/L (0–4 mg/L). (**B**,**C**) Isobologram analysis of Checkerboard assay of cells grown with increasing concentrations of WH and antibiotics. Panel B shows the effect on *S. aureus* ATCC 29213 while panel C showed the effect on *E. coli* ATCC 25922. Fractional Inhibitory Concentrations (FICs) of WH are reported on the *x*-axis and FICs of antibiotics are reported on the *y*-axis. Dotted line represented limits for considering a combination as synergistic (0.5). FIC Index (FICI) values are indicated and are colored as green if synergistic (FICI ≤ 0.5).

To test if WH is synergistic with antibiotics, Checkerboard assays were carried out on the Gram-positive bacteria *S. aureus*, which is known for its sensitivity to WH and where the effect of HAMA on quorum sensing is well-documented [22]. Checkerboard assays were also carried out on *E. coli*, a Gram-negative bacteria, where phenolic compounds like gallic acid (present in WH) were shown to inhibit bacterial growth by binding to bacterial cell membranes [30]. The sample Checkerboard assay is presented in Figure 2a, indicating synergism between WH and linezolid against *S. aureus*. Isobologram analysis (Figure 2b,c and Table 2) indicates that on *S. aureus*, WH is synergistic with chloramphenicol and linezolid, is cumulative with rifampin and vancomycin, and is indifferent to amikacin. Other combination with antibiotics against *E. coli* showed an indifferent interaction.

### 3.4. The Effect of whISOBAX and Ciprofloxacin on Staphylococcal Growth and Pathogenesis (Biofilm Formation and Toxin Production)

**Figure 3:**
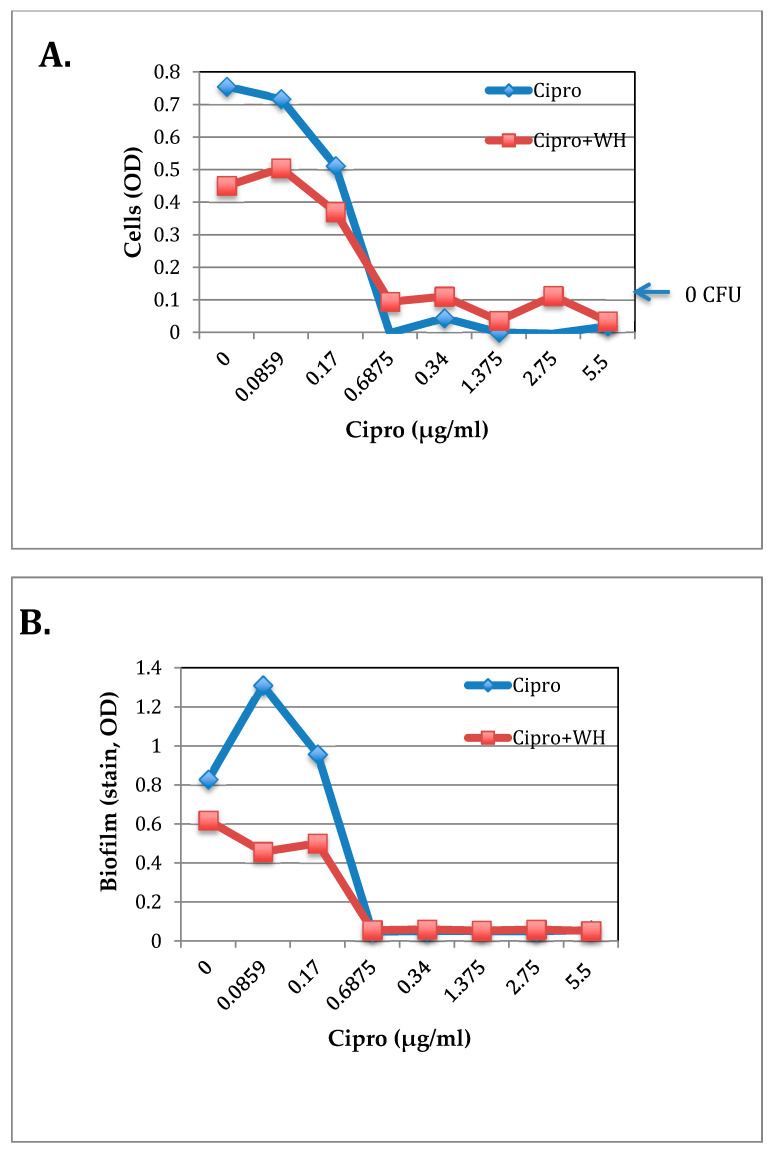

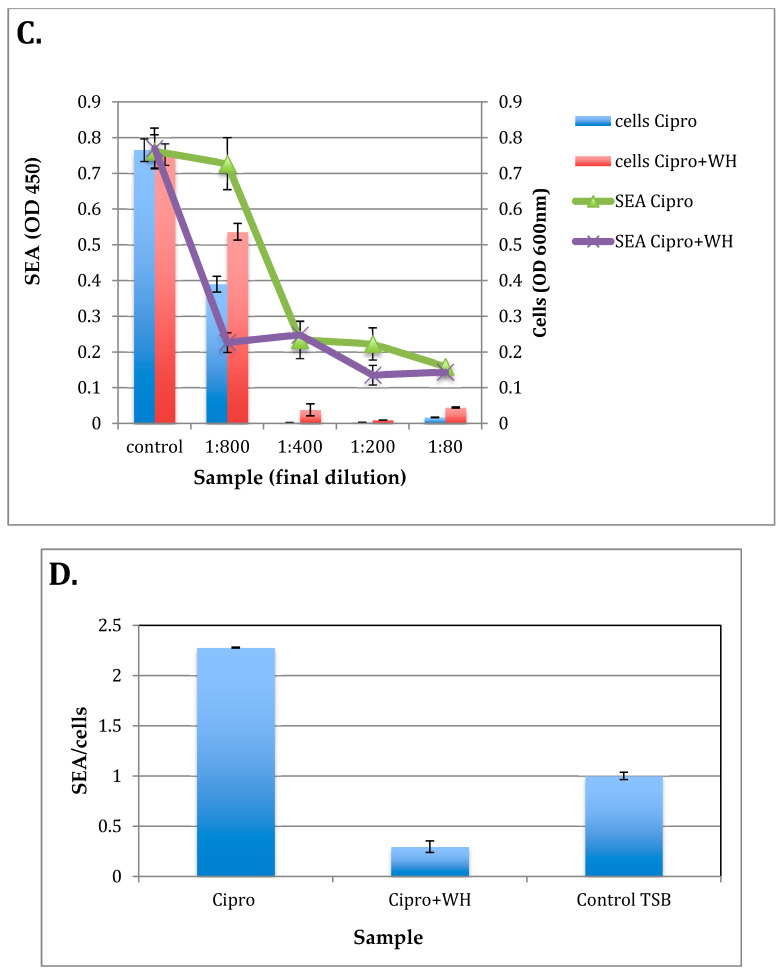
The effect of ciprofloxacin (Cipro) +/− whISOBAX (WH) on *S. epidermidis* growth and biofilm formation (**A**, **B**) and on *S. aureus* growth and toxin production (**C**, **D**). Control cells were grown without test solution. *S. epidermidis* were grown overnight at 37 °C with increasing concentrations of Cipro and/or WH, and cell density determined (**A**). Unbound cells were removed, and biofilm was stained and OD determined (**B**). *S. aureus* were grown with increasing concentrations of Cipro and/or WH, and cell density determined (**C**). Supernatants were collected, and the presence of Staphylococcal enterotoxin A (SEA) was tested by ELISA (**C**). The ration between SEA and cell density when *S. aureus* were grown in subinhibitory concentrations of Cipro is shown in (**D**), which correspond to Cipro diluted 1/800, i.e., ½ its MIC levels.

Ciprofloxacin (Cipro) is used to treat different types of bacterial infections, but at subinhibitory concentrations, it has been shown to increased toxin production in *E. coli* [31,32,33]. WH has been shown to suppress staphylococcal pathogenesis (biofilm formation and toxin production) by a Hama-mediated mechanism. To test if subinhibitory concentrations of Cipro enhance staphylococcal pathogenesis and test if the addition of WH can inhibit that, the effect of Cipro and WH were tested on staphylococcal growth and pathogenesis (biofilm formation by *S. epidermidis* and toxin production by *S. aureus*). To test for the effect of Cipro +/− WH on *S. epidermidis* growth and biofilm formation, Checkerboard Testing was carried out where *S. epidermidis* was grown overnight in TSB with increasing concentrations of Cipro (0.086–5.5 µg/mL) and/or whISOBAX (0.0025–2.5%) (initial dilution of 1/40). At the end of the overnight incubation period cell density was determined spectrophotometrically. The MBC of Cipro alone was determined to be 0.6875 µg/mL. The MBC of WH was determined to be 2.5% (1/40 dilution). The MBC of Cipro alone or in combination with WH remained the same, suggesting indifference between the two. However, cell burden was reduced up to 1.7 times in the presence of 1.25% WH (Figure 3a).

To test for biofilm formation by the cells that had been incubated with Cipro +/− WH, unbound cells were removed, and remaining attached bacteria were stained and their OD determined. As shown in Figure 3b, in the presence of subinhibitory concentrations of Cipro (below 0.017 µg/mL), biofilm formation was enhanced 1.6 times than control cells (growth without Cipro or WH), and up to 2.8 times more than when 1.25% WH was added. These results indicate that subinhibitory concentrations of Cipro exacerbate biofilm formation but WH prevents that and further reduces biofilm burden. Since biofilms contribute to antibiotic treatment failures, these results suggest that the addition of WH would be highly beneficial.

Many toxins are produced by *S. aureus*, where one of them is Staphylococcal enterotoxin A that causes food poisoning [34,35]. The effect of Cipro +/− WH was tested on the growth of *S. aureus* and on toxin (SEA) production. As shown in Figure 3b, Cipro with or without WH were equally effective in inhibiting *S. aureus* growth (MIC at WH diluted 1/400 (0.125 mg/mL) and Cipro at 0.5 µg/mL). At ½ the MIC (at 1/800), however, only Cipro + WH inhibited *S. aureus* toxin production (Figure 3c). When the amount of SEA is compared to cell density (Figure 3d), it becomes evident that while in control samples containing only TSB the ratio between SEA and cell density is one, the ratio of SEA/cells is significantly higher (*p* < 0.001) than in the presence of Cipro alone. This suggests that at concentrations below its MIC, Cipro enhances toxin production in *S. aureus*, but the addition of WH prevents that, and toxin production is repressed.

These results indicate the benefit of using WH in combination with antibiotics, to suppress not only cell growth but also pathogenesis, and prevent potential activation of toxin production in the presence of subinhibitory antibiotic concentrations.

## 4. Discussion

Diseases caused by bacteria are an ongoing challenge worldwide. Antimicrobial resistance and biofilm formation on host cells and medical devices can lead to treatment failures, and the production of bacterial toxins further worsens potential outcome [36].

Results presented here show that WH had growth inhibitory effects both on Gram-positive and Gram-negative bacteria, with dilution factors ranging from 1/10 to 1/2000 (0.05–10% WH). This suggests that efficacy may depend more on the molecular mechanisms of suppression involved (see below), like interference with quorum sensing by HAMA [22] and/or disruption of cell membrane function by gallic acid [30]. The most WH-sensitive ones tested were Staphylococci (including MRSA) and Enterococci, followed by Acinetobacter, Klebsiella, Escherichia, Pseudomonas, and streptococci. Variability in sensitivity to WH can be beneficial in targeting specific infections, with potentially less disruption to normal microflora.

Results presented here also show that effect of commonly used antibiotics can be enhanced by the addition of WH, and that WH is synergistic with antibiotics like chloramphenicol and vancomycin. These results are in agreement with previous studies indicating that WH enhances the effect of germicides like iodine-based teat dips [36].

Importantly, results presented here show that WH also acts as an inhibitor of biofilm formation by *S. epidermidis* and toxin production by *S. aureus*. This is especially important considering that subinhibitory concentrations of some antibiotics (see below) actually enhance bacterial pathogenesis.

WH is stable also when kept at higher temperatures (tested for up to 2 months at 37 °C), and contains a high amount of phenolic compounds (12.66 mg/mL GAE), where 25% of that is gallic acid, gallocatechin and catechin [24]. Gallic acid was shown to cause irreversible changes in membrane properties of both Gram-negative and Gram-positive bacteria, including a decrease in negative surface charge, and changes in cell permeability due to pore formation [30]. Gallic acid content thus allows WH to act as an extract capable of directly killing bacterial cells, and as such, WH acts like a more conventional bactericidal agent. But unique to WH is the fact that 75% of the total phenolic content is HAMA, which has specific capabilities in targeting staphylococcal pathogenesis; like its peptide analogue RNAIII inhibiting peptide (RIP), HAMA inhibits a signal transduction pathway that leads to regulation of multiple genes encoding for proteins necessary for the survival of the bacteria in the host [22,37,38,39,40,41]. This process allows for nonpathogenic bacteria that are part of the normal flora to survive. But if their numbers increase beyond normal levels and biofilms are formed and toxins are produced, such bacteria would be targeted by HAMA and their pathogenesis is suppressed. As has already been demonstrated in thousands of patients suffering from chronic wound infections involving, but not limited to, staphylococci, the use of WH or analogues helped resolve even the most persistent resistant infections [42,43].

Currently, bacterial resistance to commonly used antibiotics is increasing globally. Infections caused by antibiotic-resistant microorganisms are difficult, and sometimes impossible, to treat. In most cases, antibiotic-resistant infections require extended hospital stays, and the need to use costly or toxic alternatives. Antibiotic use can also result in secondary infections. The most common ones are antibiotic-associated diarrhea, which occurs in 10–20% of people treated with antibiotics. According to the CDC [6], the more severe cases are caused by *C. difficile*, now resulting in about 15,000 deaths a year in the US. The major risk factor for such secondary infections is taking antibiotics in the previous several weeks. High-risk antibiotics have been shown to be clindamycin, cephalosporins, and quinolones, like ciprofloxacin (see below) [6]. Combination therapy using natural compounds with antibiotics is one of the possibilities to overcome antibiotic resistance, and synergies between antibiotics and natural compounds has been demonstrated [44,45,46]. Our studies suggest a synergistic activity of WH with multiple antibiotics tested, most notably linezolid that is used against vancomycin-resistant staphylococcal strains.

Ciprofloxacin (Cipro) is a fluoroquinolone antibiotic that inhibits bacterial DNA gyrase, and is used to treat different types of bacterial infections. However, at subinhibitory concentrations, Cipro has been shown to induce toxin (stx2) production in *E. coli* EHEC O157:H7, and its use was thus not recommended in cases where toxin-producing strains are suspected [31]. The induction of toxin production by Cipro in *E. coli* has been shown to occur by triggering bacterial SOS response [32]. Similarly, as shown here, Cipro induces staphylococcal pathogenesis (biofilm formation in *S. epidermidis* and toxin production in *S. aureus*). Staphylococcal virulence has been shown to be upregulated by phosphorylation of TRAP [37], which also regulates bacterial stress response [39,40,41,47]. In the presence of WH, toxin production and biofilm formation are inhibited, thanks to the high content of HAMA, which has been shown to inhibit TRAP phosphorylation and subsequent gene regulation [22]. Put together, in the presence of HAMA (or WH), TRAP is inhibited, stress response and pathogenesis are suppressed, and bacteria’s ability to survive in the host is compromised [22,42,43]. Thus, not only can WH enhance antibacterial activity, it can also prevent pathogenesis that might be induced by certain antibiotics at subinhibitory concentrations.

Other antibiotics, like Oxacillin, which inhibit peptidoglycan synthesis, have been shown to induce the production of certain proteins in *S. aureus*, even when the net protein synthesis is inhibited [39]. It is suggested that specific enhancement in protein synthesis is an attempt by the bacteria to counter the inhibitory effects of these antibiotic. Interestingly, one of these proteins whose production is induced by Oxacillin is TRAP [48]. This would suggest a possible attempt by the bacteria to enhance its stress response and pathogenesis, which is important for its ability to survive under stressful conditions induced by the antibiotic. In the presence of WH, through a HAMA-dependent mechanism, TRAP phosphorylation is inhibited, and stress response and pathogenesis are suppressed.

## 5. Conclusions

To summarize, WH suppresses bacterial growth and pathogenesis, and thus may be able to eradicate bacterial infections also where biofilms are involved. Furthermore, WH enhances the effect of commonly used antibiotics while preventing bacterial pathogenesis, thus enabling eradication of infection while reducing the need of excessive antibiotic use.

## Figures and Tables

**Table 1:** Minimal inhibitory concentrations (MICs) of WH and respective dilution factors against various bacteria. * MIC range represents the minimum and maximum MIC values obtained in the different replicates.

Bacteria	MIC Mean (µg/mL)	MIC Range (µg/mL) *	Mean Dilutions of WH (50 mg/mL) that Inhibit Growth
**Gram Positives**			
*S. epidermidis* RP62A	26	26	1/1920
*S. aureus* ATCC 43300	45	26–78	1/1120
*S. aureus ATCC* 29213	104	104	1/460
*E. faecalis* ATCC 29212	45	39–52	1/1120
*E. faecium* 64/3	34	19–52	1/1000
*S. agalactiae* 1357	3958	1250–6667	1/23
*S. pneumoniae* ATCC49619	4583	2500–6667	1/13
**Gram Negatives**			
*A. baumannii* ATCC 19606	182	156–208	1/280
*K. pneumoniae* ATCC 700603	572	312–833	1/110
*P. aeruginosa* ATCC 27853	2916	1667–5000	1/30
*E. coli* ATCC25922	3657	1250–10,000	1/25

**Table 2:** Fractional Inhibitory Concentration Index (FICI) of WH and antibiotics. FICI was determined, where the MIC of the antibiotic in combination divided by the MIC of antibiotic alone + MIC of WH in combination divided by the MIC of WH alone was calculated. FICI ≤ 0.5 indicates synergism between compounds, >0.5 to <1.0 as cumulative interaction, >1.0 to < 4.0 as indifferent.

Antibiotic	Minimum FICI	Maximum FICI	Interaction
***S. aureus* ATCC 29213**			
Rifampin	0.625	1.250	Cumulative
Chloramphenicol	0.375	1.125	Synergistic
Amikacin	1.125	2.000	Indifferent
Vancomycin	0.625	1.500	Cumulative
Linezolid	0.375	1.250	Synergistic
***E. coli* ATCC 25922**			
Rifampin	1.125	2.000	Indifferent
Chloramphenicol	1.125	2.000	Indifferent
Amikacin	1.125	3.000	Indifferent
